# Cannabinoid interventions for improving cachexia outcomes in cancer: a systematic review and meta‐analysis

**DOI:** 10.1002/jcsm.12861

**Published:** 2021-12-08

**Authors:** Lucile Simon, Christine Baldwin, Anastasia Z. Kalea, Adrian Slee

**Affiliations:** ^1^ Division of Medicine University College London London UK; ^2^ Department of Nutritional Sciences King's College London London UK; ^3^ Institute of Cardiovascular Sciences University College London London UK

**Keywords:** Cancer, Cachexia, Cannabinoids, Appetite, Weight, Quality of life

## Abstract

Cancer‐associated cachexia (CAC) is a wasting syndrome characterized by involuntary weight loss and anorexia. Clear definition and diagnostic criteria for CAC are lacking, which makes it difficult to estimate its prevalence, to interpret research and to compare studies. There is no standard treatment to manage CAC, but previous studies support the use of cannabinoids for cachexia in other chronic diseases including HIV and multiple sclerosis. However, only a few randomized controlled trials (RCTs) and one meta‐analysis of this intervention in cancer populations are available. Non‐randomized studies of interventions (NRSIs) are often excluded from reviews due to variable methodology and potential for biases. This review aimed to consider NRSIs alongside RCTs to provide a complete summary of the available evidence that clinical decision makers could use in future investigations. Literature searches were conducted using three databases for relevant RCTs or NRSIs according to Cochrane methodology. Abstract and full texts of retrieved manuscripts were selected and retrieved by two investigators based on the PRISMA‐A guidelines, and risk of bias and quality of evidence assessments were performed. Outcome data on weight, appetite, quality of life, performance status, adverse effects, and mortality were combined by narrative synthesis and meta‐analysis where possible. Ten studies were included, four of which were RCTs and six NRSIs matching the eligibility criteria. Very low‐quality evidence from meta‐analysis suggested no significant benefits of cannabinoids for appetite compared with control (standardized mean difference: −0.02; 95% confidence interval: −0.51, 0.46; *P* = 0.93). Patient‐reported observations from NRSIs suggested improvements in appetite. Another meta‐analysis of moderate quality evidence showed that cannabinoids were significantly less efficient than active or inactive control on quality of life (standardized mean difference: −0.25; 95% confidence interval: −0.43, −0.07; *P* = 0.007). The effectiveness of cannabinoids alone to improve outcomes of CAC remains unclear. Low‐quality evidence from both RCTs and NRSIs shows no significant benefits of cannabinoids for weight gain, appetite stimulation, and better quality of life, three important outcomes of cachexia. Higher quality research integrating cannabinoids into multi‐modal therapies may offer better opportunities for developing CAC‐specific treatments. This review also highlights that findings from non‐randomized studies of interventions (NRSIs) can provide evidence of the effects of an intervention and advocate for the feasibility of larger RCTs.

## Introduction

Cachexia is a multifactorial wasting syndrome characterized by involuntary weight loss through the ongoing loss of muscle mass, with or without loss of adipose tissue.^1^, ^2^, ^3^ It is a life‐threatening aspect of advanced chronic disease, including cancer. Cancer‐associated cachexia (CAC) is driven by tumour‐host interactions resulting in progressive physical deterioration and functional impairment.^4^, ^5^, ^6^ It is associated with decreased quality of life (QoL) and tolerance to treatment and increased morbidity and mortality.^2^, ^7^, ^8^, ^9^ Attempts to define cachexia are relatively recent; therefore, estimates of prevalence vary considerably,^10^ particularly in cancer patients depending on tumour type and stage.^11^ A lack of consensus on definition and diagnostic criteria also makes it difficult to interpret research on the effectiveness of interventions and to compare studies.^2^, ^3^, ^12^

There are no standard treatments or guidelines to manage CAC,^8^ but an effective strategy should aim to reduce or prevent wasting to favour survival in advanced cancer patients.^13^, ^14^ Numerous therapeutic approaches have been developed to target wasting, weight loss and anorexia, three hallmarks of cachexia, including anti‐cytokine therapies and metabolic mediators to counter wasting (e.g. glucocorticoids, anabolic steroids, progestogens, and beta‐adrenoreceptor agonists); caloric or nutrient supplementation to prevent weight loss and promote muscle and weight gain; and using appetite stimulants like megestrol acetate and cannabinoids to manage anorexia.^15^, ^16^, ^17^ The benefits of megestrol acetate for appetite, caloric intake, nutritional status, QoL, and reduced muscle wasting in cachexia are well documented,^15^, ^18^, ^19^ but the weight gain associated with this drug often reflects fat deposition with little or no muscle growth.^17^, ^20^, ^21^ The potential of cannabinoids to relieve symptom burden in chronic diseases is recognized,^22^, ^23^ but their effectiveness in CAC is unclear.

Cannabinoids mimic the effect of human endocannabinoids on metabolism and appetite by interacting with their receptors, CB1, and CB2,^24^ and may have therapeutic benefits for body weight and appetite. The most commonly studied cannabinoids are delta‐9‐tetrahydrocannabinol (THC) and cannabidiol (CBD). Currently, only three cannabinoid‐containing drugs are commercially available for clinical use. Both Marinol and Cesamet, also known as dronabinol and nabilone respectively, are synthetic analogs of THC indicated for chemotherapy‐induced nausea and vomiting in the USA and Canada.^23^ Dronabinol is sometimes also prescribed for HIV/AIDS‐associated wasting syndrome. Sativex is a cannabis extract buccal spray containing a mixed ratio of THC and CBD adjunctively indicated for neuropathic and cancer pain.^25^ None of the above are currently indicated for CAC.

Previous randomized controlled trials (RCTs) report that cannabinoids can induce improvements in body weight, appetite, physical functioning and QoL in cachectic patients with other chronic diseases including HIV infection and multiple sclerosis.^26^, ^27^, ^28^ However, this is not well studied in cancer patients. Most reports suggesting the benefits of cannabinoids for appetite in CAC are anecdotal or lack methodological homogeneity,^29^ and few RCTs and one meta‐analysis are available.^16^ The latter suggested cannabinoids were associated with improvements to appetite but not to QoL and more adverse events compared with placebo. However, the authors included few studies, which were small and likely underpowered, provided a poor description of their methodology, and carried out no supplementary searching suggesting that studies might have been missed.

Non‐randomized studies of interventions (NRSIs) on the effect of cannabinoids on outcomes of CAC are available. To our knowledge, NRSIs have not yet been considered in a systematic review and are often excluded due to their methodological variability and the potential for biases. However, the relatively small number of RCTs is likely to give an incomplete picture and result in missing outcomes.^30^, ^31^ Findings from NRSIs could provide additional evidence on these outcomes and encourage the feasibility of larger, higher quality RCTs.^32^

Given the difficulty for clinical practitioners to manage cachexia and its severe health implications for patients, it is important to evaluate all the existing evidence relevant to developing efficient therapies. This review aimed to consider NRSIs alongside RCTs for a comprehensive approach to the available evidence on cannabinoid interventions in CAC, in order to inform clinical decisions and future investigations.

## Methods

This systematic review was conducted according to the Preferred Reporting Items for Systematic Reviews and Meta‐Analysis (PRISMA)^33^ and Cochrane Handbook for Systematic Reviews of Interventions^34^ guidelines. A protocol was developed prior to initiating the review, but not published. A research question was formulated using the PICO approach. The inclusion and exclusion criteria, outcomes, and search were planned to capture as many studies as possible. Prior to the start of the investigation, it was also agreed by the investigators that meta‐analyses would be performed where possible and narrative syntheses generated for all other outcomes.

### Eligibility criteria

#### Participants

Although the existing criteria for recognition of CAC enables more efficient diagnosis,^2^, ^3^ it is under‐recognized in clinical practice. To reflect diagnostic oversights, adult (>18 years) cancer patients, whose baseline characteristics were judged to describe cachexia, were eligible, including individuals of any gender, ethnicity, disease stage in any care setting, and undergoing chemotherapy or radiotherapy. Individuals with an eating disorder, undergoing treatment for appetite and weight loss, or with a history or current habit of marijuana use were excluded.

#### Intervention

Cannabinoid‐based interventions included any smoked or ingested medical marijuana, plant‐based cannabinoids (THC and CBD) and synthetic cannabinoids (dronabinol, nabilone, or any other pharmaceutical form).

#### Comparison

No restrictions on the comparisons were applied to allow inclusion of qualitative evidence. Treatment comparisons were any active or inactive control. Active control included nutritional interventions administered orally (food fortification, snacks, and nutrient/caloric supplementation), while pharmacological interventions and co‐interventions involved the use of active drugs (appetite stimulants, anticytokines, and metabolic mediators), and other forms of cannabis. Inactive control included placebo, standard care or no treatment.

#### Outcome measures

Primary outcomes included changes in weight and appetite and secondary outcomes included performance status (PS), quality of life (QoL), adverse events (AEs), treatment‐related side effects, and mortality. Outcomes could be patient‐reported or clinician‐reported, using continuous or discreet methods, including validated scales, questionnaires, and interviews. The rationale for selecting the above outcomes was guided by previous work^16^, ^27^ and these were selected to reflect how patients perceive the symptoms of cachexia.

#### Studies

No restrictions on study design were applied to permit a comprehensive evaluation of the outcomes in a population of advanced cancer patients, in which ethical concerns complicate methodological implementation, such as randomization or blinding. All RCTs and NRSIs were included (refer to Quality of studies and risk of bias).

### Search strategy

#### Electronic searches

To account for the lack of a standard definition of cachexia, the search strategy was designed to incorporate any terms associated with CAC, including wasting syndrome and weight loss, and cannabis‐based interventions. The electronic databases Ovid MEDLINE, Ovid Embase, and PubMed were searched from inception to May 2020, combining keyword terms with medical subject headings (MeSH), or equivalent, where possible. The full search strategies are shown in *Figure* S1. No restrictions on language or publication date and status (i.e. published, unpublished, conference abstracts, awaiting assessment, and in progress) were applied to account for the expected scarcity of evidence in this field.

#### Supplementary searching

Databases of registered and ongoing studies and reviews were searched, including PROSPERO, the ISRCTN registry, and ClinicalTrials.gov. Additional studies were identified by reference checking and citation tracking from studies identified as eligible for inclusion. Clarification was sought from corresponding authors where necessary.

### Data collection and analysis

#### Selection of studies

All references were imported into and duplicates were removed using EndNote. One investigator independently conducted the first screening to identify eligible titles and abstracts. Studies were excluded where interventions did not involve cannabis, target CAC, or report on any given outcome of interest. Any study that did not meet the inclusion criteria was excluded, while in a second screening the full texts of potentially eligible studies for inclusion were reviewed and selected, including articles in English, French, and Spanish. Any uncertainties were discussed and resolved with at least one more co‐investigator. The full text of some studies that met the eligibility criteria were unavailable online and these were not included as no response was received upon request for access and/or contacting the author. Because these may have been of value in the analysis, the description of these studies is available in *Table* S1.

### Quality of evidence and risk of bias

Two different scales, both recommended Cochrane tools, were used to assess methodological quality and risk of bias regarding study participation and attrition, measurement of the prognostic factor and outcomes, confounding, statistical analysis and reporting. ‘Risk of bias’ (Rob2) was used to assess RCTs and ‘Risk of bias in non‐randomized Studies‐of Interventions’ (ROBINS‐I) to assess NRSIs. Uncertainties were discussed and resolved and each aspect was rated. The results of both assessments were combined and summarized in a table where +, −, or ? indicated the level of risk as low, high, or unclear, respectively.

The overall quality of evidence for each outcome was assessed using the Grading of Recommendations Assessment, Development and Evaluation (GRADE) approach. The quality of evidence was downgraded for any significant study limitations (risk of bias), indirectness, important inconsistency or heterogeneity, imprecision, or potential publication bias, or upgraded for large magnitude or confounding effects, and dose–response gradient. Data from NRSIs started at low quality. The body of evidence for each outcome was judged as very low, low, moderate, or high and summarized narratively (*Table* S2). Reasons for down‐grading or up‐grading evidence are referred in the table's footnotes.

### Data synthesis and statistical analysis

Data extraction was carried out using a data collection form designed on Microsoft Office Excel (Microsoft Corporation) for this review, and data input was reviewed by two investigators. The form included extracted data on study design, participants, intervention, publication details, and outcomes of interest from the eligible studies using a template designed for this review. Where numerical data were not addressed in the full text or supplementary material and was only reported in graphs, and when no response was received from corresponding authors, it was extracted using a ruler on a magnified version of the figure.

Studies were grouped according to their design (RCTs or NRSIs). Outcome data and trends were described in terms of the number of studies, relevant effects, and statistical significance (*P* < 0.05) reported on the outcome. Results were combined narratively or by meta‐analysis where possible.

Studies only reported sufficient data to conduct meta‐analyses for QoL and appetite, which were pooled using Review Manager (RevMan version 5.4; The Nordic Cochrane Center) using a continuous, inverse variance, random effects analysis. A random effects model was used because of variability in both study design and participants, and interventions. Mean change and standard deviations (SDs) were available for most studies. Otherwise baseline and end‐of‐study means, and *P* values, were used to calculate mean change and compute SDs. In one RCT comprising of two treatment arms, data from each arm was compared with half the number in the control group to avoid duplicate reporting.^35^ The standardized mean difference (SMD) was used to account for differences in tools or methods of data collection for similar outcomes.^36^, ^37^ The inconsistency (*I* ^2^) statistic was used to assess heterogeneity, which was subsequently classified as *I* ^2^ < 40%—low; 30 to 60%—moderate; 50 to 90%—substantial and >75%—considerable.^38^

## Results

**Figure 1:**
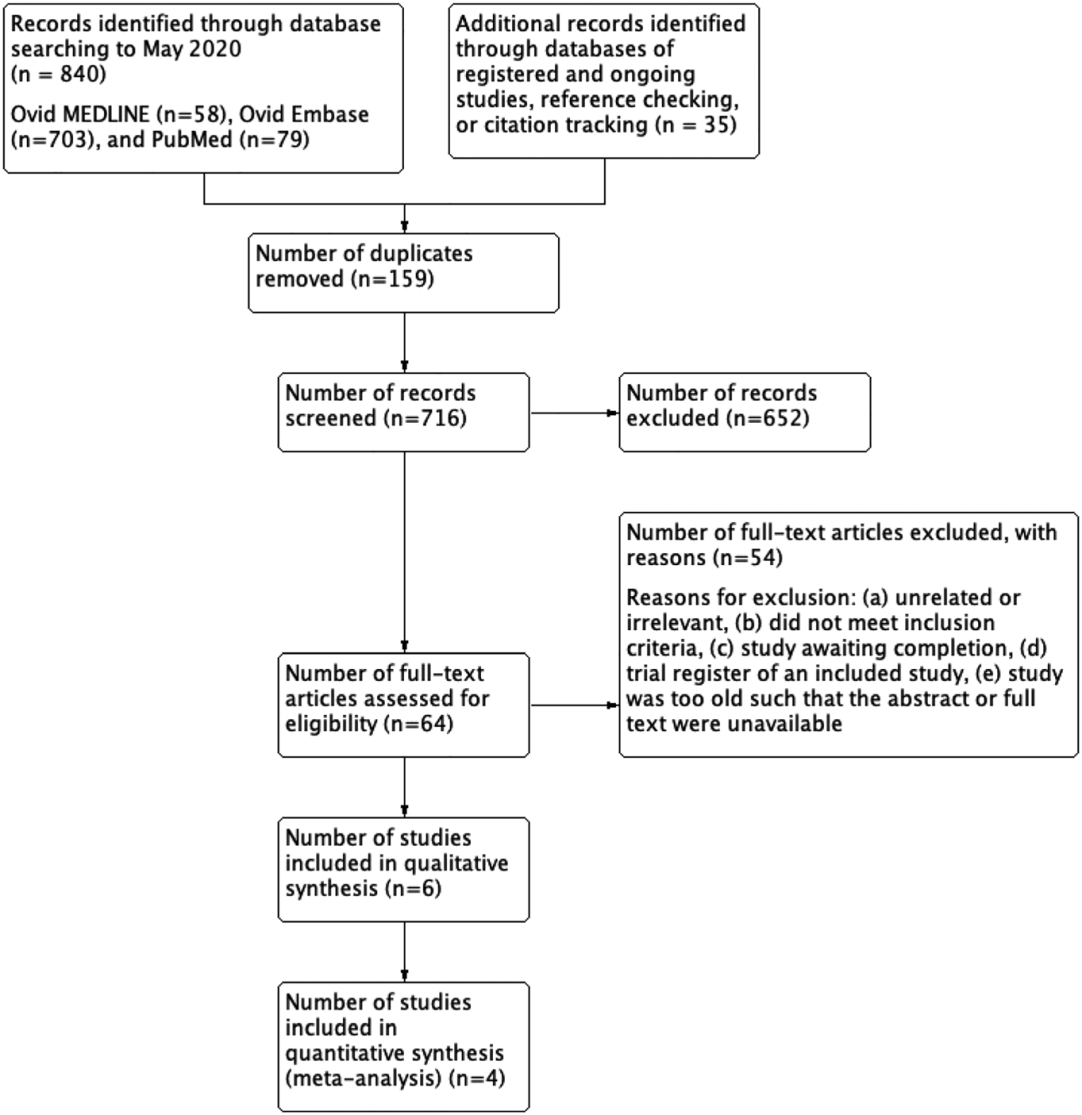
Study selection process following the PRISMA guidelines.^33^

Eight hundred and seventy‐five studies were identified from searches with 716 titles and abstracts screened for eligibility following deduplication. We obtained and scrutinized 64 full‐text papers, of which 10 were included in this review (*Figure* 1). Studies were excluded if they: were unrelated or irrelevant, did not meet the inclusion criteria, were awaiting completion, or were a registered trial of an included study. Two records were trials registered on ClinicalTrials.gov. One^39^ was an ongoing study that begun in July 2020 and is awaiting completion in October 2021 (*Table* S3). One^40^ was the ClinicalTrials.gov record reported by an included study. Six studies (*Table* S1) were excluded because the full text could not be obtained online or retrieved physically.

### Characteristics of included studies

Ten studies were included in this review^23^, ^35^, ^41^, ^42^, ^43^, ^44^, ^45^, ^46^, ^47^, ^48^ (*Table* 1). All participants were adult cancer patients, mean age ranging from 47.3 to 67 years. The presence of cachexia was confirmed as previously described, using guidance from existing criteria. Study duration varied from 18 days to 6 months, including both intervention and follow up. Two studies^42^, ^48^ were open‐label continuation studies. Methods of outcome reporting varied and comprised a range of both validated scales and procedures as described in *Tables* S5–S8.

**Table 1:** Characteristics of included studies

Summary of studies									
RCTs									
Study author, year	Study design	Duration and follow up	Participant characteristics	Sample size	Intervention	Route	Comparator	Outcome of interest	Additional outcomes
Brisbois *et al*., 2011^41^	RCT, Canada	18 days; F/U: 30 days	12 male, 9 female; mean age (SD): Intervention group: 67(10.9) years Comparator group: 65.5(8.0) years Advanced cancer with decreased food intake	I: 24 C: 22	2.5 mg THC: once daily for 3 days; before bedtime for first 2 days and before supper on third day) twice daily on fourth day (1 before lunch, 1 before dinner) option to increase to 20 mg/day	Oral	2.5 mg placebo: once daily for 3 days; before bedtime for first 2 days and before supper on third day) twice daily on fourth day (1 before lunch, 1 before dinner) option to increase to 20 mg/day	Appetite QoL	Total calorie and macronutrient intake Nausea Food preference Chemosensory alterations
Jatoi *et al*., 2002^42^	RCT, United Kingdom	open‐label continuation; ‘patient continued on treatment for as long as they and their healthcare providers thought it beneficial or until toxic side effects prompted study withdrawal’	Intervention group: 65% male, 35% female; mean age (SD): 65(11) years Comparator group: 66% male, 34% female; mean age (SD): 67(10) Advanced cancer with self‐reported weight loss >5 lbs (2.3 kg) in last 2 months, loss of appetite, <20 kcal/kg intake per day, and 0–2 PS score	I: 152 C: 159	2.5 mg dronabinol capsules twice daily plus liquid placebo	Oral	800 mg megestrol acetate liquid suspension daily plus capsule placebos	Weight Appetite QoL	Side effects
Strasser *et al*., 2006^35^	RCT, Germany	6 weeks; F/U at week 2, week 4 and week 6	54% men, 46% women; mean age: 61 years Advanced cancer with involuntary weight loss >5% in last 6 months, anorexia, and <2 PS score	I: 100 (THC); 95 (cannabis extract, CE) C: 48	THC: 2.5 mg THC capsulesCE: 2.5 mg: 1 mg THC:CBD capsules Three times 2‐weeks supply taken twice daily (1 hour before lunch and dinner, or at bedtime), preferably with milk	Oral	Placebo capsules containing a standardization medium Three times 2‐weeks supply taken twice daily (1 hour before lunch and dinner, or at	Body weight Appetite change QoL change Adverse events	Other symptoms Functional domains of QoL
Turcott *et al*., 2018^43^	RCT, Mexico	8 weeks; F/U at week 2, 4 and 8	Intervention group: 3 male (21.4%), 11 female (78.6%); mean age (SD): 61.1(10.6) years; 6 moderately malnourished, 8 severely malnourished Comparator group: 4 male (21.1%), 15 female (78.9%); mean age (SD): 52.6(11.8) years mean age; 6 moderately malnourished, 13 severely malnourished Confirmed NSCLC with anorexia and <2 PS score	47 (33 included in analysis)	0.5 mg nabilone (CESAMET) for 2 weeks Increased to 1 mg for next 6 weeks	Oral	0.5 mg placebo for 2 weeks Increased to 1 mg for next 6 weeks	Weight change Appetite change HRQL	Biochemical parameters Nutritional consumption
Non‐RCTs									
Bar‐Sela *et al*., 2019^44^	Pilot study	6 months	62.5% male, 38.5% female; median age: 66; median weight: 65.5 kg Advanced cancer with weight loss of >5% in last 2 months, loss of appetite and <3 PS score	11	10 mg THC:CBD (9.5:0.5) or 5 mg THC:CBD (4.75:0.25) cannabis capsules Once daily for 2 weeks, then twice daily (first in the morning, then after 8 hours)	Oral	None	Weight QoL	Tolerance to cannabis dosage
Kasvis *et al*., 2019^45^	Retrospective observational study	120 days; F/U at 30–75 days and 75–120 days (clinic visits)	Cancer patients referred to the Cannabis Pilot Project from McGill University Health Centre Mean age (SD): 61 (11) years; 49% male, 51% female; 43% anorexia	37	medical cannabis treatment based on individual assessment by multidisciplinary team	Not specified	None	Weight improvement Appetite improvement	
Kasvis *et al*., 2019^46^	Retrospective chart review	3 months	Mean age (SD): 47.3(16.1) years; 34 male (63%), 20 female (37%); 23 cancer (42.6%), 31 non‐cancer (57.4%);	54 (51 included in analysis)	cannabinoid therapy (number of participants): THC/CBD (1:1): 6 total participants with SC: 2 participants no SC: 4 participants THC‐rich: 17 total participants with SC: 8 participants no SC:9 participants CBD‐rich: 0 participants combined therapies: THC/CBD and THC‐rich: 17 total participants with SC: 7 participants no SC: 10 participants THC/CBD and CBD‐rich: 7 total participants with SC:3 participants no SC: 4 participants THC‐ and CBD‐rich: 17 total participants with SC: 8 participants no SC: 9 participants THC/CBD, THC‐rich, CBD‐rich: 1 total participant with SC: 0 participants no SC: 1 participant	20.4% oral, 25.9% inhaled, 53.7% combined oral and inhaled	None	Weight Appetite	
Nelson *et al*., 1994^47^	Phase II trial	28 days: F/U at week 2 and 4	13 male, 6 female; mean age: 65.12 years, median (range) age 64(52–81) years; median (range) PS: 2(0–3); median (range) mini‐mental status exam score: 29 (13–30); Advanced cancer patients	10	THC 2.5 mg p.o. t.i.d. one hour after meals 2.5 mg b.i.d. for 3 days if >65 years	Oral	None	Unclear	
Plasse *et al*., 1991^23^	Non‐RCT	3 and 6 weeks	33 male, 9 female; Karfnofsky performance status (median, range): 80 (60–100); previous THC exposure: 4 Cancer patients	42	I: dronabinol treatment, 4 groups; group 1: 2.5 mg q.d. group 2: 2.5 mg b.i.d. group 3: 5 mg q.d. group 4: 5 mg b.i.d. study 1: group 3 received dose before breakfast; study 2: group 3 received dose before dinner;	Oral	None	Weight change Appetite (visual analog scales and scores)	Mood
Walsh *et al*., 2005^48^	Case series	Open continuation; F/U biweekly or at every outpatient clinic visit until considered stable, then per routine clinical practice	Patients treated chronically with escalating dronabinol doses for cancer‐related anorexia	6	dronabinol was titrated from 7.5 to 15 mg daily in 5 patients, 1 patient remained on initial dose;	Oral	None	Weight change Self‐reported appetite	Self‐reported food intake Efficacy Side effects

Abbreviations: b.i.d., twice daily; C, comparison; CBD, cannabidiol; CE, cannabis extract;; F/U, follow up; HRQL, health‐related quality of life; I, intervention; NSCLC, non‐small cell lung cancer; p.o., oral; PS, performance status; q.d., daily; QoL, quality of life; RCT, randomized controlled trial; SC, synthetic cannabinoid; SD, standard deviation; THC, tetrahydrocannabinol; t.i.d., three times daily.

Four studies were RCTs (*n* = 647) assessing the effect of cannabinoids on at least one outcome (appetite, weight, or QoL) in advanced cancer patients and two^41^, ^43^ were pilot studies. Three multi‐centre trials^35^, ^41^, ^42^ used dronabinol and one study^43^ used nabilone. Three RCTs^35^, ^41^, ^43^ used a placebo as the control. The remaining study, a large RCT,^42^ used megestrol acetate plus placebo as the standard treatment arm, dronabinol capsules plus liquid placebo as the intervention arm, and a combination of both dronabinol and megestrol acetate in a third intervention arm. Only the standard treatment and first intervention arm were included in this analysis, because they both included placebo and were most comparable. One study^35^ included a third intervention arm with cannabis extract (CE) to compare with THC, which were individually compared with the comparison arm (placebo). Two RCTs^35^, ^42^ prescribed 2.5 mg THC doses twice daily for the entire treatment and two^41^, ^43^ prescribed increasing doses (from 0.5 mg to 20 mg per day maximum).

The remaining six studies were NRSI (*n* = 157) assessing the effects of cannabis or cannabinoid treatment on appetite and weight in cancer patients. Participants' physical status and disease stage, and study design, varied. Where specified, treatment was administered orally. Two were retrospective studies^45^, ^46^ one of which one^45^ was only reported as a conference abstract. The other^46^ was a retrospective chart review where one of the following cannabinoid therapies were either taken orally or inhaled: 1:1 THC/CBD, THC‐rich, CBD‐rich, 1:1 THC/CBD + THC‐rich, 1:1 THC/CBD + CBD‐rich, THC‐ + CBD‐rich, or all three. Two were single‐arm intervention studies^23^, ^44^ one of which included two consecutive studies (lasting 3 and 6 weeks, respectively). In this study, patients were assigned to one of four treatment groups: 2.5 mg once or twice daily or 5 mg once or twice daily. Patients on 5 mg once daily received their dose before breakfast in the first 3 weeks, then before dinner until the end of the 6 weeks. One was a Phase‐II trial^47^ and one was a case series^48^ both of which specifically aimed to treat cancer‐related anorexia.

### Risk of bias and quality assessment

**Figure 2:**
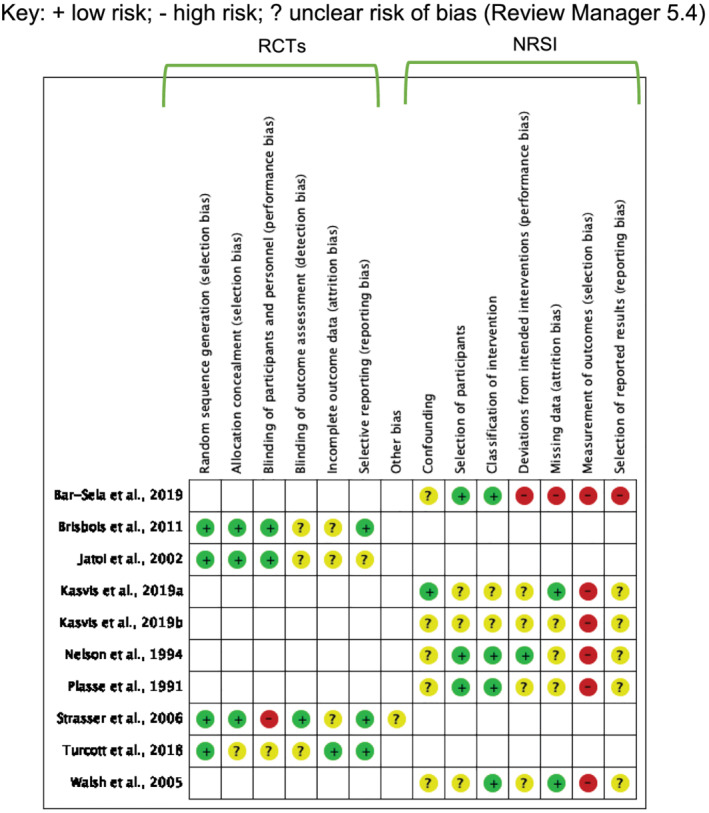
Risk of bias summary: review authors' judgements of risk of bias for each included study key: + low risk; − high risk; ? unclear risk of bias (Review Manager 5.4).

Risk of bias was assessed for all outcomes of interest for which both objective and subjective measures were used in data collection methods (*Figure* 2). Risk of bias was determined as unclear by the author wherever information was lacking or vague. All four RCTs were at low risk of selection and reporting bias due to appropriate randomization, allocation, and analyses methods. One^35^ was at high risk of performance bias due to major protocol violations by 84 participants, and one was unclear^43^ because allocation was carried out by the protocol coordinator. Low or unclear risk of bias in the remaining three domains was determined primarily due to uncertainties concerning how missing data, and withdrawals were handled.

The remaining studies were at unclear risk of performance or reporting bias due to uncertainties. They also all had unclear risk of confounding for not controlling for pre‐conceptions associated with cannabis intake (i.e., cravings, relaxation and sleepiness related to the THC‐induced high, a bad trip or other associated side effects), recall bias, and no controls.

Risk of classification of intervention was judged to be low^23^, ^44^, ^47^, ^48^ or unclear^45^, ^46^ on the basis that the nature of the intervention was reported in sufficient detail. There was low^46^, ^48^ or unclear^23^, ^45^, ^47^ risk of attrition bias due to unexplained missing outcome data. All studies were at high risk of selection bias because interventions were not blinded. One study^44^ was also at high risk of performance, attrition, and reporting bias due to significant withdrawals, no explanation or evidence of adjustments or sensitivity analyses, and insufficient information.

### Primary outcomes

#### Weight

Eight of ten studies (80%), two RCTs and six NRSIs, reported data on aspects of this outcome (*Table* 2). The method of assessing weight varied and included self‐reported or physician‐reported, mean baseline and end‐of‐study body weight, body weight change (gain or loss) reported as a percentage, mean or median, median rate of weight loss, and as percentage of patients with >10% weight gain.

**Table 2:** Summary of findings from studies reporting on weight

Outcome (units)	Study author, year	Intervention	Sample size	Results		
				Intervention	Control	p value
RCTs						
% patients with >10% increase above BL	Jatoi *et al*., 2002^42^	2.5 mg dronabinol capsules b.i.d.	I: 152 C: 159	Self‐reported 3, physician‐reported 5	Self‐reported 11, physician‐reported 14	0.02, 0.009
% patients with a max weight gain of 0%, 1–4%, 5–9% or >10%				65, 23, 8, & 3	57, 23, 10, & 10	0.041
Mean change in body weight, kg (SD)	Turcott *et al*., 2018^43^	0.5 mg nabilone for 2 weeks, then 1 mg for 6 weeks	I: 14 C: 19	−1.4 (1.6)	−1.09 (2.6)	0.724
NRSI						
Percent % change, range	Bar‐Sela *et al*., 2019^44^	Cannabis capsules 10 mg THC:CBD (9.5:0.5) or 5 mg THC:CBD (4.75:0.25) q.d. for 2 weeks then b.i.d. (morning, then +8 h)	11	7.7–21.6	None	Not reported
Mean BL and final weight, kg (SD) Mean weight change, kg	Kasvis *et al*., 2019^45^	medical cannabis treatment based on individual assessment by multidisciplinary team	37	BL: 70.7 (19.3) Final: 66.1 (23.0) Calculated from mean BL and final weight: −4.6	None	0.509
Mean BL and final weight, kg (SD) Mean weight change, kg	Kasvis *et al*., 2019^46^	cannabinoid therapy (# of participants): THC/CBD (1:1) (6 total) THC‐rich (17 total) CBD‐rich (0 total) combined therapies (# of participants): THC/CBD and THC‐rich (17 total) THC/CBD and CBD‐rich (7 total) THC‐ and CBD‐rich (17 total) THC/CBD, THC‐rich, CBD‐rich (1 total)	54 (51 included in analysis)	BL: 70.7 (14.6) Final: 71.0 (14.8) Calculated from mean BL and final weight: 0.3	None	Not reported
Median weight change, kg (range)	Nelson *et al*., 1994^47^	THC 2.5 mg p.o. t.i.d. 1 h post‐meals	6	1.3 (1.0–2.7)	None	Not reported
Descriptive weight change, n		2.5 mg b.i.d. for 3 days if >65 years		3 gained weight 2 maintained a stable weight 1 lost weight	None	N/A
Median rate of weight loss before and after therapy, kg/months	Plasse *et al*., 1991^23^	I: dronabinol treatment, 4 groups; group 1: 2.5 mg q.d. group 2: 2.5 mg b.i.d. group 3: 5 mg q.d. group 4: 5 mg b.i.d. Study 1: group 3 received dose before breakfast; Study 2: group 3 received dose before dinner;	42	Before I: group 1: −3.2 group 2: −3.2 group 3: −1.5 group 4: −1.1 After I: group 1: −2.3 group 2: −1.5 group 3: −1.4 group 4: 0.2	None	Group 1 and group 3 only: <0.05
Median weight gain, kg (range)	Walsh *et al*., 2005^48^	7.5 to 15 mg dronabinol q.d. in 5 patients, 1 patient remained on initial dose	5	1 (0.5)	None	Not reported
Individual patient weight change, patient #, kg			6	1: +1 2: 0 3: n/a (withdrew) 4: +5 5: +1 6: +3	None	Not reported

Abbreviations: b.i.d., twice daily; C, comparison; CBD, cannabidiol; CE, cannabis extract; F/U, follow up; I, intervention; NRSI, non‐randomized study of intervention; q.d., daily; RCT, randomized controlled trial; SC, synthetic cannabinoid; SD, standard deviation; THC, tetrahydrocannabinol; t.i.d., three times daily.

One RCT^42^ found the standard treatment, megestrol acetate, resulted in greater weight gain (*P* = 0.02). The other RCT with placebo as a control reported no difference in mean (SD) change in weight between groups.^43^ Four of six NRSIs^23^, ^44^, ^46^, ^47^ reported small improvements to weight in groups receiving cannabinoids. Improvements ranged from 0.3 kg mean weight change,^46^ 1.0–1.3 kg median weight gain,^47^, ^48^ and 7.7–21.6% increase in weight.^44^

One of the two remaining NRSI reported a reduction in weight in groups receiving cannabinoids,^45^ and the other reported a smaller rate of weight loss in groups receiving higher doses of dronabinol (*P* < 0.05).^23^ The quality of evidence was very low.

#### Appetite

All studies (100%) reported on this outcome. Appetite was assessed using a range of different methods including validated scales,^23^, ^35^, ^41^, ^43^, ^44^, ^45^, ^46^ validated questionnaires^42^, ^47^ and self‐evaluations.^48^

**Figure 3:**
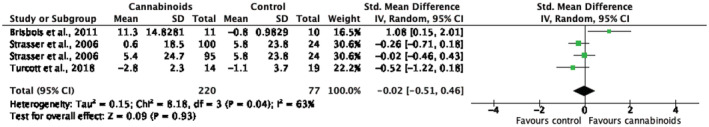
Meta‐analysis of the effect of cannabinoids on change in appetite in patients with cancer cachexia.

Three RCTs^35^, ^41^, ^43^ (*n* = 297) reported data in a format suitable for pooling in a meta‐analysis (*Figure* 3, *Table* S4). There was no difference in change in appetite in groups receiving cannabinoid treatment compared with groups receiving placebo, standard mean difference (SMD): −0.02 [95% confidence interval (CI): −0.51, 0.46; *P* = 0.93]. Heterogeneity was substantial (*I* ^2^ = 63%, *P* = 0.04). A sensitivity analysis revealed that when the study favouring intervention^41^ was excluded, *I* ^2^ was reduced to 0% and there remained no difference between groups. This study had a small number of participants, used a different tool to evaluate appetite and reported much greater changes in score, drawing attention to the validity of the methods and subsequent results.

Data from the remaining seven NRSIs (*Table* 3) were not suitable for a meta‐analysis due to insufficient information and absence of comparison. In two of the RCTs,^35^, ^42^ more patients in the control group reported improved appetite compared with the intervention group, with one^42^ reporting a significantly greater improvement in appetite in the group receiving megestrol acetate. All six NRSIs reported a positive effect on appetite post‐treatment, three of which reported a significant improvement^23^, ^45^, ^46^ The remaining three observed fewer complaints about appetite loss^44^ and better ratings of appetite, calorie count, and food intake^47^ from patients, although statistical significance was not reported. The quality of evidence was very low.

**Table 3:** Narrative summary of findings from studies reporting on appetite

Study ID	Method of data collection and sample size	Outcomes
RCTs		
Jatoi *et al*., 2002^42^	Best follow‐up response (%) from validated questionnaires completed at BL and 1 month I: 159 C: 152	More patients in the control group vs. the intervention group reported: Increased appetite after vs. before illness (*P* = 0.0005) Increased food intake after vs. before illness (46% vs. 25%; *P* < 0.0001) ‘Very good’ appetite (21% vs. 11%; *P* = 0.001) Appetite ‘increased very much’ after vs. before intervention (16% vs. 8%; *P* < 0.05) Eating ‘very much more’ due to medication (15% vs. 5%; *P* = 0.008) Better tasting food (51% vs. 27%; *P* = 0.0003) Increased food intake with medication (65% vs. 44%; *P* = 0.002)
Strasser *et al*., 2006^35^	Appetite Loss categoric scale in the EORTC QLQ‐C30^49^ I: THC: 100 CE: 95 C: 48	Increased appetite: 60% THC patients, 75% CE patients group and 72% control group (*P* = 0.068)
NRSI		
Bar‐Sela *et al*., 2019^44^	Appetite Loss subscale in the EORTC QLQ‐C30 before and at the end of the study I: 6 C: none	Significantly fewer complaints about appetite loss post‐treatment (*P* = 0.05) Increase in appetite 2 weeks post‐treatment Individual scores before and after treatment (*n*: before, after): 1: 0, 0 2: 32, 32 3: 78, 32 4: 78, 0 5: 78, 0 6: 100, 32
Kasvis *et al*., 2019^45^	Revised ESAS^50^ questionnaire completed at baseline, Visits 1 and 2 I: 37 C: none	75% reported improvements in anorexia at Visit 1 Significant mean (SD) improvement in appetite over F/U (3.5 (3.0), 2.2 (2.4) and 1.5 (2.2); *P* = 0.033)
Kasvis *et al*., 2019^46^	ESAS questionnaire, repeated at 3 months F/U I: 54 C: none	Mean (SD) appetite score improved significantly from BL to F/U [5.07 (3.21) and 3.56 (3.15); *P* = 0.0026]
Nelson *et al*., 1994^47^	3‐Q interview, 1 appetite‐related Q: Since starting this drug, has your appetite shown no improvement, shown slight improvement, shown major improvement, or become completely normal? I: 18 C: none Weekly 1 day food diary recorded by the patient I: 19 C: none	13 patients reported improved appetite (Spearman's rank correlation, *r* = 0.16) 10 reported ‘slight improvement’ 3 reported ‘major improvement’ Calorie count: Improved in 8 patients Did not change in 1 patient Not reported in 10 patients Median (range) increase: 1032 (574–2436) kcal/day
Plasse *et al*., 1991^23^	VAS completed before each meal with defining terms ‘extremely hungry’ and ‘not hungry at all’ I: 42 C: none	General increase in median appetite score (mm) from BL to end of study for each group (*P* < 0.05 between Groups 1 and 3 and 1 and 4): Group 1: −5 Group 2: 16 Group 3: 2 Group 4: 3
Walsh *et al*., 2005^48^	Self‐reported subjective evaluation 5‐Q biweekly interview in outpatient clinics I: 6 C: none	‘Rated their food intake as better or the same’ ‘Maintain stable appetite despite progressive disease’ Question 1: How is your appetite? Better: 3 patients Same: 3 patients Question 2: How is your food intake? Better: 2 patients Same: 4 patients

Abbreviations: BL, baseline; C, comparison; CE, cannabis extract; EORTC‐QLQ‐C30, European Organisation for Research and Treatment of Cancer—Quality of Life Questionnaire—Core 30; ESAS(‐r), Edmonton Symptom Assessment System (‐Revised); F/U, follow up; I, intervention; RCTs, randomized controlled trials; THC, tetrahydrocannabinol; VAS, visual analogue scale.

### Secondary outcomes

#### Performance status

Two NRSIs (20%) reported on PS (*Table* 4). There were no changes in median PS pre‐ and post‐treatment in one study.^47^ Two participants in a case series discontinued treatment due to worsened PS.^48^ The quality of evidence was very low.

**Table 4:** Narrative summary of findings from studies that reported on PS and QoL

Study ID	Method of data collection and sample size	Outcomes reported
NRSI reporting on PS		
Nelson *et al*., 1994^47^	ECOG PS score I: 19	Median (range) at: BL: 2 (0–3) Post‐study score: 2 (1–3)
Walsh *et al*., 2005^48^	Self‐reported subjective evaluation of PS I: 6	2 patients discontinued because of worsened PS
NRSI reporting on QoL		
Bar‐Sela *et al*., 2019^44^	EORTC QLQ‐C30 version 2 at Day 1 I: 6 C: none	Reported no difference before and after intervention
Walsh *et al*., 2005^48^	Self‐reported subjective measure of well‐being I: 5 C: none 5‐question interview at every outpatient clinic visit (biweekly), rated as B (better), W (worse), S (the same) or N (no) I: 6 C: none	‘Improved or remained stable’ Question 3: How do you feel overall? 3 patients reported feeling better 2 patients reported feeling the same 1 patient reported feeling worse Question 4: How is your energy level? 3 patients reported better energy levels 2 patients reported the same energy levels 1 patients reported worse energy levels

Abbreviations: C, comparison; ECOG, Eastern Cooperative Oncology Group; EORTC‐QLQ‐C30, European Organisation for Research and Treatment of Cancer, Quality of Life Questionnaire, Core 3; ESAS, Edmonton Symptom Assessment System; I, intervention; NRSI, non‐randomized study of intervention; RCT, randomized controlled trial; SD, standard deviation.

#### Quality of life

Six of ten studies (60%), four RCTs and two NRSIs, reported on this outcome with QoL being assessed using both validated scales^35^, ^41^, ^42^, ^43^, ^44^ and self‐evaluations.^48^ Where QoL instruments were used, a higher score was indicative of a higher Global QoL, and a greater change in score was indicative of a greater improvement.^51^, ^52^

**Figure 4:**
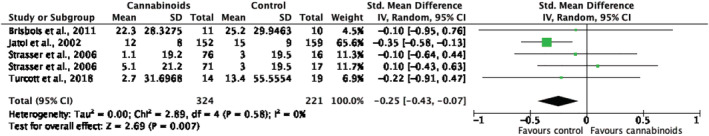
Meta‐analysis of the effect of cannabinoids on changes in quality of life (QoL) in patients with cancer cachexia.

There was sufficient data on Global QoL from four RCTs (*n* = 545) to allow a meta‐analysis (*Figure* 4, *Table* S5). There was a small and significantly greater improvement in Global QoL in groups receiving either active (megestrol acetate) or inactive (placebo) control compared with groups receiving cannabinoids, suggesting that cannabinoid treatment was less efficacious, SMD: −0.25 (95% CI: −0.43, −0.07); *P* = 0.007). There was no heterogeneity (*I* ^2^ = 0%, *P* = 0.58).

Data from the remaining two NRSIs (Table 4) were not suitable for a meta‐analysis due to insufficient data or the absence of a comparison group. Bar‐Sela *et al*.^44^ reported no difference between pre‐ and post‐intervention and Walsh *et al*.^48^ described relative improvements or stability in perceived well‐being and energy levels.

Data from one study^46^ reporting QoL in a wider patient population not treated with cannabinoids (*Table* S6) was excluded from the analysis because it did not match the inclusion criteria for intervention. The quality of evidence was moderate.

#### Adverse events

Nine of ten studies (90%), four RCT and five NRSI, reported on this outcome (*Table* S7). Assessment of AEs included number or percentage of patient‐reported symptoms or events, loss of follow up related to cancer, number of withdrawals or drop‐outs, and evaluation of side effects.

Two of the RCTs^41^, ^42^ showed no significant difference for the number or severity of AEs and serious AEs (SAEs), or the incidence of side effects, in the intervention compared with the control group. One^41^ reported four AEs and one SAE were possibly related to treatment. The other two RCTs^35^, ^43^ showed no significant effect, although one^35^ reported more AEs in the intervention compared with the control group.

Twenty AEs were likely to be treatment‐related, although the intervention group was twice as numerous as the control group.

Of the five NRSI, one^44^ reported withdrawals due to treatment‐related effects. This same study also reported positive secondary effects of treatment on pain and fatigue reduction, and sleep and mood improvement after intervention. Most patients in four NRSI reported side effects,^23^, ^46^, ^47^, ^48^ although it is unclear if those are related to the intervention. Only one study^48^ reported no new problems and treatment tolerability. The quality of evidence was very low.

#### Mortality

Three RCTs (30%) reported on mortality^35^, ^42^, ^43^ noting that more participants died in the intervention group compared with the control group. In one RCT,^42^ participants in the intervention group lived longer overall than participants in the control group (*Table* S8). The number of deaths in each study was small, and the quality of evidence for this outcome was very low.

## Discussion

Cachexia is wasting condition, which seriously threatens patient prognosis. Currently, there is not a standardized treatment, and the lack of a robust definition hinders our ability to conduct high‐quality research. Various therapeutic approaches have been investigated, but their effectiveness in reducing and preventing weight loss and anorexia, two key indicators of cachexia, varies. This systematic review and meta‐analysis evaluated the effect of cannabinoid‐based interventions in CAC patients for changes in body weight, appetite, QoL, PS, AEs, and mortality. The aim was to compare evidence from NRSIs alongside RCTs to provide a thorough summary of the available evidence. To our knowledge, this is the first review to include NRSIs in its analysis.

Results from six NRSIs and four RCTs were comparable to a previously published systematic review of RCTs.^16^ In this review we showed it is unclear that cannabinoids alone can induce significant improvements in weight, appetite, quality of life, performance status, adverse effects, or mortality in CAC populations.

Weight and appetite loss caused by CAC are associated with a poorer prognosis,^6^ and have become common therapeutic targets in research. Previous studies report a beneficial effect of cannabinoids for weight and appetite in elderly and chronically ill patients.^26^, ^53^, ^54^ Very low quality evidence suggested no such benefits on weight in cancer patients, independently of the study design. This is consistent with a study showing no weight change for dronabinol compared with other appetite‐stimulating medications^55^ and a systematic review reporting no significant weight change in cancer patients taking cannabis.^27^ One NRSI^23^ found a significant reduction in rate of weight loss, suggesting that cannabinoids may be more useful in delaying weight loss rather than reversing it.

Other pharmacological interventions, such as megestrol acetate and ghrelin, focused on reduced appetite and food intake resulting from weight loss.^15^, ^19^ Very low‐quality evidence suggests no significant improvements in appetite and a meta‐analysis showed that cannabinoids had no greater efficacy than a control treatment. There was high heterogeneity (*I* ^2^ = 63%, *P* = 0.04) and a small number of participants, so that further larger trials are needed. One of the included RCTs found that megestrol acetate led to greater weight gain and significant improvements in appetite,^42^ further discouraging any therapeutic advantage of cannabinoids without better‐quality studies. These findings contradict two previous systematic reviews that reported a small benefit.^16^, ^27^ The inclusion of NRSIs in the analysis may explain this disparity. Also, previous work by Wang *et al*.^16^ concluded a significant effect of the intervention only after excluding the study that reported lower rate of increased appetite in the intervention group, without providing a clear rationale.

Other publications support the benefits of cannabinoids for food intake and appetite in healthy populations and HIV patients.^27^, ^56^, ^57^ Future research should invest how the pathophysiology of CAC may inhibit this effect in cancer patients. For example, chronic cannabis use was associated with decreased food intake and a lower prevalence of obesity in two representative US surveys.^58^ Because obesity and CAC share a pathophysiology characterized by systemic inflammation and metabolic disturbances, it could be interesting to investigate if and how these mechanisms interfere in the normal stimulatory effect of cannabinoids. In particular, if they act on the human CB1 and CB2 receptors, known to act on regulatory pathways of appetite and metabolism.

Although the majority of studies used validated methods, the body of evidence was rated as very low due to methodological limitations including unmasked allocation and outcome assessment. For example, the known effects of cannabis, such as the ‘munchies’ (sudden and strong desire for food) could have influenced any patient‐perceived improvements in appetite, food intake, calorie count, and/or taste of food. Further studies on this outcome are warranted.

The benefits of cannabinoids on QoL are elusive because both across RCTs and NRSIs, the evidence is anecdotal or self‐reported. The only systematic review evaluating this outcome concluded contradictory results, where cannabinoids both have a potent and counterproductive effect on QoL.^16^ Other findings, including those in the present review, remain inconclusive.^59^ Evidence of moderate quality found no significant benefits of cannabinoids on QoL, while a meta‐analysis favoured an effect in the control group. Unlike for appetite, data from NRSIs also concluded no effect. Our analysis aligns with other publications that cannabinoids may be less efficient to improve QoL.^27^, ^60^ This effect was independent of study design, supporting the informative value NRSI may bring to RCT data.^30^, ^61^

The importance of QoL in cancer care is reported elsewhere in the literature.^62^, ^63^, ^64^ A qualitative study analysing patient perception of cannabis use emphasized that marijuana and its derivatives were increasingly popular among cancer patients to alleviate treatment‐related side effects.^65^ Any therapy for advanced chronic disease should consider the patients' lived experience of that treatment together with its efficacy. Higher quality studies using objective measurements of QoL are needed to determine the benefits of cannabinoids and provide optimal support to patients.

Other outcomes of interest included PS, AEs, and mortality. Very low‐quality evidence did not allow a conclusion on the effects of cannabinoids for PS or mortality, but suggested that the incidence of AEs was unlikely to be related to cannabinoid treatment. It was concerning that only three studies reported on mortality, despite a study finding a significant correlation between severe weight loss and time from cancer diagnosis to death.^66^

Nutritional intake was reported in some studies, but excluded from this review to avoid wrongfully equating it to appetite.^67^ However a cross‐sectional observational study finding a significant association between malnutrition and QoL in cancer patients^64^ suggested that it may be a more robust measure of appetite. Other measures include endocrine markers of appetite such as insulin and ghrelin, which were shown to be influenced by cannabis in healthy cannabis users.^68^ Together with nutritional intake, they may offer a more objective alternative to measure the orexigenic potential of cannabinoids in CAC.

### Strengths

A thorough search strategy was implemented across three databases without any limits on study design, date, language, or publication status. Compared with previous published work, this review used the most recent evidence available from both RCTs and NRSIs across more outcomes. A meta‐analysis was conducted which challenged or confirmed previous findings from other meta‐analyses.^16^, ^27^ An assessment of the quality of evidence was performed to distinguish the importance of outcomes and minimize bias towards positive results from low quality evidence.

### Limitations

The Cochrane Library was not searched because previous work by Wang *et al*.,^16^ which this review only aimed to extend, had already examined the collection. As the largest collection of medical literatures, this remains a limitation which could add value to the present study.

The identification of studies was limited by the absence of a clear definition of cachexia and lack of standardized treatment, so that cachexia was not always clearly recognized in the study populations. In the context of this review, a research project submitted to University College London, which did not allow for a double screening, only one investigator conducted the first screening. This is a major limitation implying that some relevant studies may have been excluded or missed.

The majority of the available evidence consisted of observational reports lacking a comparison group or a robust methodology, most likely due to the measurement of subjective outcomes. The lack of methodological homogeneity, expected from the inclusion of NRSI, could only be minimized where a meta‐analysis was possible. More than half the studies included were unmasked and at high risk of selection of bias, which was reflected in very low‐quality ratings of the evidence overall. The low number of studies did not permit an evaluation of publication bias via a funnel plot.

Other limitations included short study duration and small sample size undermine the informative value of this review for clinical decision makers.

## Conclusion

With no high‐quality evidence, no recommendations can be made to support the use of cannabinoids alone to improve symptoms and outcomes in CAC patients. Multi‐modal therapies integrating cannabinoids alongside other treatment strategies may have a greater potential. Without better data, identifying and investigating other nutritional and pharmacological interventions may provide more conclusive findings.

To our knowledge, our systematic review is the first to include NRSI in its method and analysis. With respect to study design, this review uniquely shows that lower quality of evidence exists in both randomized and non‐randomized studies, and that data from non‐randomized studies bring valuable information to that of RCTs. This method proved particularly useful to address a complex, challenging aspect of cancer like cachexia. Future research adapting data from both RCT and NRSI will be essential to identify interventions that address both the pathophysiological and patient‐centred aspects of cachexia, including nutritional status, appetite, and quality of life.

## Conflict of interest

The authors declare no potential conflicts of interest.

## Funding

The submission charges were funded by UCL Library.

## Ethics statement

The authors of this manuscript certify that they comply with the ethical guidelines for authorship and publishing in the *Journal of Cachexia, Sarcopenia and Muscle*.^69^ This systematic review is based on published data and does not contain sensitive clinical study or patient data.

## Supporting information


**Table S1.** Bibliography of studies unavailable in full‐text.
**Table S2.** Summary of findings and quality of evidence assessment.
**Table S3.** Ongoing clinical trial awaiting completion in October 2021.
**Table S4.** Summary of findings and meta‐analysis from studies reporting on appetite.
**Table S5.** Summary of findings and meta‐analysis from studies reporting on QoL.
**Table S6.** Summary of findings for health‐related quality of life in a population of cancer patients not treated with cannabinoids.
**Table S7.** Narrative summary of findings from studies reporting on AEs.
**Table S8.** Narrative summary of findings from studies reporting on mortality.
**Figure S1.** Search strategy for electronic databases and other resources.
