# Supplementary material for: Cannabinoid interventions for improving cachexia outcomes in cancer: a systematic review and meta‐analysis
Source: J Cachexia Sarcopenia Muscle. 2021 Dec 8;13(1):23–41. doi: 10.1002/jcsm.12861 (PMC8818598; doi:10.1002/jcsm.12861)
Supplement: Supplementary file 1 — Table S1. Bibliography of studies unavailable in full‐text. Table S2. Summary of findings and quality of evidence assessment. Table S3. Ongoing clinical trial awaiting completion in October 2021. Table S4. Summary of findings and meta‐analysis from studies reporting on appetite. Table S5. Summary of findings and meta‐analysis from studies reporting on QoL. Table S6. Summary of findings for health‐related quality of life in a population of cancer patients not treated with cannabinoids. Table S7. Narrative summary of findings from studies reporting on AEs. Table S8. Narrative summary of findings from studies reporting on mortality. Figure S1. Search strategy for electronic databases and other resources. [file JCSM-13-23-s001.docx]

**Supporting Information**

**Table S1** Bibliography of studies unavailable in full-text

| Author, year | Study title | Reference type | Description and location | Intervention | Outcomes | Author’s comments |
| --- | --- | --- | --- | --- | --- | --- |
| No author, 1998 [1] | Products to safely increase lean muscle mass | Newspaper article in Positive Health News | Report of THC-containing Marinol promoting appetite and an increase in body mass.  United States | N/A | - Appetite promotion - Increased body mass | This newspaper article may refer to relevant studies and provide useful background on past research. |
| Abrams, 1994[2] | Donald Abrams’ Marijuana Study | Newspaper article in Critical Path AIDS Project | Prospective randomised community-based clinical trial evaluating the effect of marijuana vs dronabinol to reduce symptoms of HIV-related wasting syndrome.  United States | High-, medium- or low-potency smoked marijuana vs dronabinol | - Appetite stimulation - Reduced weight loss - Quality of life - Adverse effects | This study does not meet our inclusion criteria but includes relevant condition (wasting) and outcomes for comparison. |
| Gorter, 2004[3] | Experiences with dronabinol (delta-tetrahydrocannabinol) in oncological patients with anorexia-cachexia syndrome. Illustration of clinical problems and therapy based on 2 case reports. | Case reports | Unavailable  Germany | N/A | - N/A | This study may report relevant observations for the effect of dronabinol on cachectic patients. |
| Maida et al., 2008 [4] | Adjunctive nabilone in cancer pain and symptom management: a prospective observational study using propensity scoring | Prospective observational study | Assessment of the effectiveness of adjuvant nabilone (Cesamet) therapy in managing pain and symptoms in 112 advanced cancer patients during a 30-day follow-up period.  Canada | Cesamet (adjuvant nabilone) | - Differences in ESAS pain scores between treated and untreated patients - Differences in morphine-sulfate-equivalent use | The study noted changes in appetite relevant for comparison in this review. |
| Wilson et al., 2007[5] | Anorexia of aging in long term care: is dronabinol an effective appetite stimulate?—a pilot study | Pilot study in the Journal of Nutrition, Health and Aging | Retrospective observational study examining the effect of a 12-week course of dronabinol in 28 long-term care facility patients with anorexia and significant weight loss. | Dronabinol | - Body weight | This study reports changes in body weight related to intervention, but it could not be included in our review because the abstract does not indicate if subjects are cancer patients or other. |

**Table S2** Summary of findings and quality of evidence assessment

| Population: adult cancer cachexia patients  Setting: any (hospital, elderly care, home)  Intervention: any cannabis-based intervention addressing cachexia, or cachexia symptoms or related proxies (e.g. nutritional intake for appetite) in cancer patients  Comparison: active or inactive control | | | |
| --- | --- | --- | --- |
| Outcome | Participants (studies) | Quality of evidence (GRADE) | Cannabinoids vs control, SMD (95% CI) |
| Weight | 497 (8) | + Very low^a,b,c,d,e^ | Two studies noted a significant effect of cannabinoids on a parameter of weight change, but one was at high risk of selection bias.  Two studies reported no difference, and four reported minor changes without assessing statistical significance. Most studies were at unclear risk of bias, unmasked and had no comparison or control group. |
| Appetite  Meta-analysis  Narrative analysis | 297 (3)  718 (8) | + Very low^b,c,d,f^ | -0.02 (-0.51 to 0.46)  Eight studies reported data that could not be pooled. All reported a positive effect in the intervention group compared to the control group, but only four assessed statistical significance. Most studies measured appetite using validated methods but were at unclear risk of bias, unmasked and had no comparison or control group. |
| PS | 25 (2) | + Very low^a,b,c,d,e,g^ | One study reported no change and one study reported 2 withdrawals due to worsened PS, but both had very small sample sizes in which allocation was uncertain. |
| QoL  Meta-analysis  Narrative analysis | 545 (4)  12 (2) | +++ Moderate^b^ | **Favoured control** -0.25 (-0.43 to -0.07)  One study reported no difference, and one reported improvement, but neither assessed statistical significance nor had a comparison. Allocation concealment was uncertain and risk of selection bias was high in both studies. |
| AEs | 640 (9) | + Very low^a,b,c,d,g,h^ | None of the studies specified the validity of the methods used to assess this outcome.  Five reported treatment related side effects, but only two assessed statistical significance. Four also reported side effects but it was unclear if those were treatment related. Half the studies were unmasked, at unclear risk of bias and had no control group. |
| Mortality | 587 (3) | + Very low^d,e,g^ | Two studies noted more deaths in the intervention group than in the control group, while one showed the opposite effect. None assessed statistical significance. |
| *Abbreviations: C – comparison; CI – confidence interval; I – intervention; PS – performance status; SD – standard deviation; SMD – standard mean difference; THC – tetrahydrocannabinol;*  GRADE (Grading of Recommendations, Assessment, Developments and Evaluation) Working Group grades of evidence:  High quality: Further research is very unlikely to change our confidence in the estimate of effect.  Moderate quality: Further research is very likely to have an important impact on our confidence in the estimate of effect and may change the estimate. Low Quality: Further research is very likely to have an important impact on our confidence in the estimate of effect and is likely to change the estimate.  Very low quality: Any estimate of effect is uncertain | | | |
| a – evidence was from studies with unclear risk of bias across several domains b – allocation was uncertain and selection bias is likely or high  c – design and methodological limitations (no comparison / control)  d – small sample size  e – small number of studies  f – large effect size (wide CI) and/or overlapping CI and/or substantial unexplained heterogeneity g – insufficient information  h – no clear or appreciable effect (benefit or harm) | | | |

**Table S3** Ongoing clinical trial awaiting completion in October 2021

| Author, year | Study design | Duration and follow up | Participant characteristics | Sample size | Intervention | Route | Comparator | Outcome of interest | Additional outcomes |
| --- | --- | --- | --- | --- | --- | --- | --- | --- | --- |
| Chasen, 2020 [6] | RCT, United States | 12 weeks | Adult (>18) male and female advancer cancer patients with weight loss and grade 2 cachexia, or grade 3 cachexia, in last 6 months | N/A | Cannabinoid-based medication (PPP011):   - 1 capsule inhaled 3 times daily with a vaporizer device | Inhaled | Placebo:   - 1 capsule inhaled 3 times daily with a vaporizer device | - Physical functioning related to advancer cancer - Cachexia | - Pain - Patient nutritional and functional assessment |

**Table S4.** Summary of findings and meta-analysis from studies reporting on appetite

| Outcome (units) | Study  Author, year | Method and sample size (included in analysis) | Results | | |
| --- | --- | --- | --- | --- | --- |
|  |  |  | **Mean (SD)** | **SMD (95% CI)** | **p value** |
| Change in score (mm) | Brisbois et al., 2011 [8] | 100mm Satiety Labeled Intensity Magnitude [9]  I: 11  C: 10 | I: 11.3 (14.8)  C: -0.8 (1.0) | 1.08 (0.15 – 2.01) | 0.03 |
| Increase in score (mm) | Strasser et al., 2006 [10] | Visual analog scale [11]  I:   - THC: 100 - CE: 95   C: 48 | I:   - THC: 0.6 (18.5) - CE: 5.4 (24.7)   C: 5.8 (23.8) | THC vs C: -0.26 (-0.71 – 0.18  CE vs C: -0.02 (-0.46 – 0.43) | THC: 0.95  CE: 0.46 |
| Change in score (mm) | Turcott et al., 2018 [12] | Visual analog scale  I: 14  C: 19 | I: -2.8 (2.3)  C: -1.1 (3.1) | -0.52 (-1.22 – 0.18) | 0.219 |
| *Abbreviations: C – comparison; CE – cannabis extract; CI – confidence interval; I – intervention; SD – standard deviation; SMD – standard mean difference; THC – tetrahydrocannabinol;* | | | | | |

**Table S5** Summary of findings and meta-analysis from studies reporting on QoL

| Outcome (units) | Study  Author, year | Method and sample size (included in analysis) | Results | | |
| --- | --- | --- | --- | --- | --- |
|  |  |  | **Mean (SD)** | **SMD (95% CI)** | **p value** |
| Change in global QoL score | Brisbois et al., 2011 [8] | Functional Assessment of Anorexia/Cachexia Therapy [13]  I: 11  C: 10 | I: 22 (28.3)  C: 25.2 (29.9) | -0.10 (0.95 – 0.76) | 0.026 |
| Change in UNISCALE score | Jatoi et al., 2002  [14] | UNISCALE [15]  I: 152  C: 159 | I: 12 (8)  C: 15 (9) | -0.35 (-0.58 – -0.13) | 0.19 |
| Change in composite score of global health status and QoL | Strasser et al., 2006 [10] | EORTC-QLQ-C30  I:   - THC: 71 - CE: 76   C: 33 | I:   - THC: 5.1 (21.2) - CE: 1.1 (19.2)   C: 3 (219.5) | THC vs C: -0.10 (-0.64 – 0.44)  CE vs C: -0.10 (-0.43 – 0.63) | THC: 0.43  CE: 0.80 |
| Change in global health status and QoL score | Turcott et al. 2018 [12] | EORTC-QLQ-C30  I: 14  C: 19 | I: 2.7 (31.6)  C: 13.4 (55.5) | -0.22 (-0.91 – 0.47) | 0.755 |
| *Abbreviations: C – comparison; CE – cannabis extract; CI – confidence interval; EORTC-QLQ-C30 – European Organisation for Research and Treatment of Cancer – Quality of Life Questionnaire – Core 30; I – intervention; QoL – quality of life; SD – standard deviation; SMD – standard mean difference; THC – tetrahydrocannabinol.* | | | | | |

**Table S6** Summary of findings for health-related quality of life in a population of cancer patients not treated with cannabinoids

| Summary of findings from Kasvis et al b – reporting on quality of life for all cachexia patients | | |
| --- | --- | --- |
| Study  Author, year | Method of data collection and sample size | Outcomes reported |
| Kasvis et al., 2019 [7] | Single-item FWB score in the ESAS questionnaire  I: 512  C: none | Mean (SD):   - RC (n=71): 6.07 (0.33) - C (n=154): 4.8 (0.2) - PC (n=115): 4.7(0.3) - NC (n=172): 4.2(0.2)   Comments:   - RC had significantly greater poor sense of wellbeing than other cachexia stages (p<0.05) - Fatigue was predictive of poor wellbeing in C and RC |
| *Abbreviations: C – comparison; C – cachexia; EORTC-QLQ-C30 – European Organisation for Research and Treatment of Cancer – Quality of Life Questionnaire – Core 30; ESAS – Edmonton Symptom Assessment System; I – intervention; NC – no cachexia; PC – pre-cachexia; RC – refractory cachexia; SD – standard deviation;* | | |

**Table S7** Narrative summary of findings from studies reporting on AEs

| Study  Author, year | Method of data collection and sample size | Outcomes reported |
| --- | --- | --- |
| RCTs | | |
| Brisbois et al., 2011 [8] | Side Effect Survey  11-point ESAS to assess nausea  Patient-reported side effects  I: 11  C: 10 | Quality of sleep and relaxation were more frequently reported to be pleasant. No other difference in survey responses were reported (p>0.05).  Nausea scores were unaffected by the intervention (p=0.532)  AEs that occurred:   - Intervention group: nausea, hives/rash, shortness of breath/fluid on lungs, stomach cramps, tiredness/drowsiness, pain, diarrhea, headache, dehydration, pneumonia, seizure, unsteady feet, low blood count, irregular heartbeat, thrush, oedema, vaginal discharge and trouble sleeping - Control group: nausea, hives/rash, bowel obstruction, shortness of breath/fluid on lungs, stomach cramps, tiredness/drowsiness, pain, dehydration, pneumonia, confusion and fever   Most AEs were unrelated to THC therapy, 6 were unclear and 4 were possibly related  Most SAEs were unrelated to THC therapy, 4 were unclear and 1 (irregular heartbeat) was possibly related to treatment;   - 6 patients in the intervention group and 6 in the control group discontinued treatment due to SAEs   No difference between treatment arms during the trial or in the 30- day F/U for the number of AEs and SAEs (p=0.622 and p=0.244 respectively)  THC was well tolerated |
| Jatoi et al., 2002 [14] | Patient reported side effects (%)  I: 152  C: 159 | More male participants reported impotence in the control group than in intervention group (18% vs 4%, p=0.002)  Otherwise the incidence of side effects (including vomiting, fluid retention, muddled thinking, drowsiness, loss of coordination and inappropriate behaviour was not statistically significant.  The severity of nausea, vomiting, neurocortical dysfunction, edema, ascites, pleural effusion or thrombosis was not statically different. |
| Strasser et al., 2006 [10] | Number of reported side effects  I:   - THC: 100 - CE: 95   C: 48 | A total of 526 AEs were reported:   - 197 AEs were reported in the THC group - 238 AEs were reported in the CE group - 91 AEs were reported in the control group   AEs that occurred more than 10 times included nausea, fatigue, pain, anemia, dizziness, dyspnea, diarrhea and obstipation.  AEs were unrelated or unlikely to be for 415 AEs, probably for 90 AEs and likely for 20 AEs   - 1 AEs was undetermined - 1 SAE was unexpected   More AEs occur in the intervention group than in the control group |
| Turcott et al., 2018 [12] | Loss of follow up related to cancer  I: 14  C: 19 | In intervention group:   - 2 participants required hospitalisation - 2 participants’ clinical condition deteriorated   In control group:   - 2 participants required hospitalisation - 5 participants’ clinical condition deteriorated |
| NRSI | | |
| Bar-Sela et al., 2019 [16] | Number of withdrawals  Evaluation of side effects  I: 6  C: none | - 4 patients withdrew from the study in the first 2 weeks to treatment-related side effects - 2 patients withdrew from the study between 2 weeks and 4.5 months due to side effects of cannabis intake   Side effects included tiredness, dizziness, disorientation, anxiety, hallucinations, and altered general function.  All side-effects occurred 1 to 2 hours after capsule intake, lasted for 2 to 3 hours and caused incapacity to be physically active |
| Kasvis et al., 2019 [7] | Patient reporting side effects (%)  I: 54  C: none | - 20.4% of participants reported mild side effects - 75% reported no side effects - 3.7% did not report |
| Nelson et al., 1994 [17] | Patient-reported side effects  I: 19  C: none  Number of withdrawals and reason  I: 10  C: none  Types of side effects  I: 19  C: none | 15 participants reported side effects, 4 did not  3 participants withdrew with grade I side effects  3 participants withdrew due to decreasing PS  1 participant withdrew to begin radiation treatment  1 participant withdrew and refused to answer questions  1 participant experienced grade I slurred speech  3 participants experienced grade I nausea) |
| Plasse et al., 1991 [18] | Number of dropouts and reason  I: 42 | 10 patients dropped out due to side effects, including dizziness, memory, and mood changes |
| Walsh et al., 2005 [19] | Self-reported subjective evaluation  5-question interview at every outpatient clinic visit (biweekly), rated as B (better), W (worse), S (the same) or N (no)  I: 6  C: none | ‘all tolerated dronabinol without toxicity’  Question 5 : Do you have any new problems?   - All patients answered no |
| *Abbreviations: AE – adverse event; C – comparison; ESAS – Edmonton Symptom Assessment System; I – intervention; NRSI – non-randomised study of intervention; ; SAE – serious adverse events; THC – tetrahydrocannabinol* | | |

**Table S8** Narrative summary of findings from studies reporting on mortality

| Study  Author, year | Method of data collection and sample size | Outcomes reported |
| --- | --- | --- |
| RCTs | | |
| Jatoi et al., 2002 [14] | Percentage of death  Survival in days  I: 152  C: 159 | - More participants died in the control group than in the intervention group (22% vs 15%) - Participants in the intervention group lived longer than in the control group (141 days vs 123 days) |
| Strasser et al., 2006 [10] | Number of deaths  I:   - THC: 100 - CE: 95   C: 48 | - More participants died in either intervention group than in the control group (THC: 6, CE: 4, C: 1 deaths) |
| Turcott et al., 2018 [12] | Number of deaths  I: 14  C: 19 | - More patients died in the intervention group than in the control group at the 8-week follow up (3 vs 1 deaths) |
| *Abbreviations: C – comparison; CE – cannabis extract ; I – intervention; THC – tetrahydrocannabinol;* | | |

## **Figure S1** Search strategy for electronic databases and other resources

Search terms were mapped and combined to suggested MeSH terms. Individual searches were carried out then associated as described below. All subheadings were included, unless otherwise stated.

1. Ovid MEDLINE (58 titles identified)

Cachexia/ (5176 titles identified)

OR Wasting Syndrome/ (1230 titles identified)

AND Receptor, Cannabinoids, CB1/ OR Cannabinoids/ OR Cannabis/ OR Dronabinol/ OR Cannabidiol/ OR Cannabinoid Receptor Agonists (24682 titles identified)

1. Ovid EMBASE (703)

Two separate searches were carried out using different approaches to combine terms. Both were imported into the reference manager and deduplicated.

Search 1 (647 titles identified)

cachexia/ OR wasting syndrome OR/ chronic wasting disease/ OR muscle atrophy/ OR emaciation/ OR body weight loss (84279 titles identified)

AND cannabis/ OR cannabinoid/ OR dronabinol/ OR cannabidiol/ OR cannabidiol derivative (51968 titles identified)

Search 2 (56 titles identified)

cachexia/ OR wasting syndrome/ OR chronic wasting disease (18508 titles identified)

AND muscle atrophy/ OR emaciation/ OR body weight loss (65123 titles identified)

AND cannabis/ OR cannabinoid/ OR dronabinol/ OR cannabidiol/ OR cannabidiol derivative (51968 titles identified)

1. PubMed (79 titles identified)

((“Cachexia” [MeSH Terms]) OR (“Wasting Disease, Chronic” [MeSH Terms] OR “Wasting Syndrome” [MeSH Terms]))

AND

(“Cannabinoids” [MeSH Terms] OR “Cannabinol” [MeSH Terms] OR “Cannabidiol” [MeSH Terms] OR “Receptors, Cannabinoid” [MeSH Terms] OR “Receptor, Cannabinoid, CB2” [MeSH Terms] OR “Receptor, Cannabinoid, CB1” [MeSH Terms] OR “Cannabinoid Receptor Modulators” [MeSH Terms] OR “Cannabinoid Receptor Agonists” [MeSH Terms] OR “Medical Marijuana” [MeSH Terms] OR “Endocannabinoids” [MeSH Terms] OR “Cannabis” [MeSH Terms] OR “Cannabaceae”

1. PROSPERO (0 studies identified)

cannabinoids/ OR cannabinoid receptor agonist/ OR cannabinoid receptor modulators/ OR dronabinol/ OR cannabidiol/ OR cannabis

AND cachexia/ OR wasting syndrome/ OR wasting disease, chronic

1. ISRCTN registry (0 studies identified)

Two separate searches were carried out to use alternative phrasing describing the intervention.

Text search: cannabinoids

Condition: cachexia

Text search: cannabis

Condition: cachexia

1. ClinicalTrials.gov (2 identified)

Search cachexia with cannabinoids

**References**

1. Products to safely increase lean muscle mass. Posit Health News. 1998(No 17):26.

2. Donald Abrams' marijuana study. Crit Path AIDS Proj. 1994(No 30):9-17.

3. Gorter RW. [Experiences with dronabinol (delta-tetrahydrocannabinol) in oncological patients with anorexia-cachexia syndrome. Illustration of clinical problems and therapy based on 2 case reports]. Schmerz. 2004;18 Suppl 2:S31-3.

4. Maida V, Ennis, M.,Irani, S.,Corbo, M.,Dolzhykov, M. Adjunctive nabilone in cancer pain and symptom management: a prospective observational study using propensity scoring. J Support Oncol. 2008;6(3):119-24.

5. Wilson MM, Philpot, C.,Morley, J. E. Anorexia of aging in long term care: is dronabinol an effective appetite stimulant?--a pilot study. J Nutr Health Aging. 2007;11(2):195-8.

6. Chasen M. Safety and Efficacy of Inhaled Synthetic THC/CBD for Improving Physical Functioning and for Modulating Cachexia Progression in Patients With Advanced Cancer and Associated Cachexia 2020 [Available from: <https://ClinicalTrials.gov/show/NCT04001010>.

7. Kasvis P, Vigano M, Vigano A. Health-related quality of life across cancer cachexia stages. Ann Palliat Med. 2019;8(1):33-42.

8. Brisbois TD, de Kock IH, Watanabe SM, Mirhosseini M, Lamoureux DC, Chasen M, et al. Delta-9-tetrahydrocannabinol may palliate altered chemosensory perception in cancer patients: results of a randomized, double-blind, placebo-controlled pilot trial. Annals of Oncology. 2011;22(9):2086-93.

9. Cardello AV, Schutz HG, Lesher LL, Merrill E. Development and testing of a labeled magnitude scale of perceived satiety. Appetite. 2005;44(1):1-13.

10. Strasser F, Luftner D, Possinger K, Ernst G, Ruhstaller T, Meissner W, et al. Comparison of orally administered cannabis extract and delta-9-tetrahydrocannabinol in treating patients with cancer-related anorexia-cachexia syndrome: a multicenter, phase III, randomized, double-blind, placebo-controlled clinical trial from the Cannabis-In-Cachexia-Study-Group. Journal of Clinical Oncology. 2006;24(21):3394-400.

11. Flint A, Raben A, Blundell JE, Astrup A. Reproducibility, power and validity of visual analogue scales in assessment of appetite sensations in single test meal studies. Int J Obes Relat Metab Disord. 2000;24(1):38-48.

12. Turcott JG, Del Rocío Guillen Núñez M, Flores-Estrada D, Oñate-Ocaña LF, Zatarain-Barrón ZL, Barrón F, et al. The effect of nabilone on appetite, nutritional status, and quality of life in lung cancer patients: a randomized, double-blind clinical trial. Support Care Cancer. 2018;26(9):3029-38.

13. Ribaudo JM, Cella D, Hahn EA, Lloyd SR, Tchekmedyian NS, Von Roenn J, et al. Re-validation and shortening of the Functional Assessment of Anorexia/Cachexia Therapy (FAACT) questionnaire. Qual Life Res. 2000;9(10):1137-46.

14. Jatoi A, Windschitl HE, Loprinzi CL, Sloan JA, Dakhil SR, Mailliard JA, et al. Dronabinol versus megestrol acetate versus combination therapy for cancer-associated anorexia: a North Central Cancer Treatment Group study. J Clin Oncol. 2002;20(2):567-73.

15. Ballatori E, Roila F, Ruggeri B, Bruno A, Tiberti S, Orio FD, editors. The Uniscale Assessment of Quality of Life: Applications to Oncology2010.

16. Bar-Sela G, Zalman D, Semenysty V, Ballan E. The Effects of Dosage-Controlled Cannabis Capsules on Cancer-Related Cachexia and Anorexia Syndrome in Advanced Cancer Patients: Pilot Study. Integr Cancer Ther. 2019;18:1534735419881498.

17. Nelson K, Walsh D, Deeter P, Sheehan F. A phase II study of delta-9-tetrahydrocannabinol for appetite stimulation in cancer-associated anorexia. J Palliat Care. 1994;10(1):14-8.

18. Plasse TF, Gorter RW, Krasnow SH, Lane M, Shepard KV, Wadleigh RG. Recent clinical experience with dronabinol. Pharmacol Biochem Behav. 1991;40(3):695-700.

19. Walsh D, Kirkova J, Davis MP. The efficacy and tolerability of long-term use of dronabinol in cancer-related anorexia: a case series. J Pain Symptom Manage. 2005;30(6):493-5.
